# Aminoethoxyvinylglicine and 1-Methylcyclopropene: Effects on Preharvest Drop, Fruit Maturity, Quality, and Associated Gene Expression of ‘Honeycrisp’ Apples in the US Mid-Atlantic

**DOI:** 10.3390/plants13172524

**Published:** 2024-09-08

**Authors:** Emily Johnson, Macarena Farcuh

**Affiliations:** Department of Plant Science and Landscape Architecture, University of Maryland, College Park, MD 20742, USA; emilykj@umd.edu

**Keywords:** ethylene, skin blush, plant growth regulators, transcript accumulation, fruit

## Abstract

Preharvest fruit drop is one of the main challenges in apple production as it can lead to extensive crop losses in commercially important cultivars including ‘Honeycrisp’. Plant growth regulators, such as aminoethoxyvinylglicine (AVG) and 1-methylcyclopropene (1-MCP), which hinder ethylene biosynthesis and perception, respectively, can control preharvest fruit drop, but an assessment of their effects in ‘Honeycrisp’ fruit grown under US mid-Atlantic conditions is lacking. In this study, we evaluated the effects of AVG (130 mg a.i. L^−1^) and 1-MCP (150 mg a.i. L^−1^) on preharvest fruit drop, ethylene production, fruit physicochemical parameters, skin color, and transcript accumulation of ethylene and anthocyanin-related genes in ‘Honeycrisp’ apples throughout on-the-tree ripening. We showed that both AVG and 1-MCP significantly minimized preharvest fruit drop with respect to the control fruit. Additionally, AVG was the most effective in decreasing ethylene production, downregulating ethylene biosynthesis and perception-related gene expression, and delaying fruit maturity. Nevertheless, AVG negatively impacted apple red skin color and exhibited the lowest expression of anthocyanin-biosynthesis-related genes, only allowing apples to reach the minimum required 50% blush at the last ripening stage. Conversely, 1-MCP-treated fruit displayed an intermediate behavior between AVG-treated and control fruit, decreasing ethylene production rates and the associated gene expression as well as delaying fruit maturity when compared to the control fruit. Remarkably, 1-MCP treatment did not sacrifice red skin color development or anthocyanin-biosynthesis-related gene expression, thus exhibiting > 50% blush one week earlier than AVG.

## 1. Introduction

‘Honeycrisp’ is one of the top sales cultivars in the US, but it is largely susceptible to preharvest fruit drop, reaching losses of up to 50% of the total crop [1,2]. Preharvest fruit drop generally precedes horticultural maturity starting as early as four weeks prior to the anticipated commercial harvest date [3,4]. Growers have previously relied on harvesting fruit early to avoid preharvest fruit drop, but fruit quality and storability are negatively impaired, leading to decreased fruit yield and marketable crop [5]. On the other hand, holding fruit on the tree beyond their anticipated harvest date can potentially boost crop value [6]. Hence, decreasing preharvest fruit drop is fundamental for prolonging the apple harvest and reducing losses.

Ethylene, which is known to be crucial in apple fruit ripening and quality [7], has also been reported to strongly influence apple preharvest fruit drop [8,9]. In fact, ethylene can increase fruit drop via enhancing cell wall and intercellular material breakdown in the pedicel abscission zone [10,11]. Although apple fruit display a climacteric fruit behavior (i.e., increase ethylene production and respiration rates during ripening [12,13]), ethylene production rates differ amongst apple cultivars, with higher ethylene-producing cultivars displaying increased fruit drop with respect to lower ethylene producers [14,15]. In fact, cultivars with high ethylene production, such as ‘Honeycrisp’, ‘Golden Delicious’, and ‘McIntosh’, have been shown to present an increased fruit drop, while conversely, cultivars with low ethylene production, such as ‘Empire’ or ‘Fuji’, exhibit less fruit drop [1,2,4,9,16]. Furthermore, exogenous ethylene applications in cultivars with low preharvest fruit drop rates have been reported to significantly increase fruit abscission [17], highlighting the importance of fruit ethylene production regulation as a strategy to control preharvest fruit drop. 

Plant growth regulators, such as aminoethoxyvinylglicine (AVG) and 1-methylcyclopropene (1-MCP), which hinder ethylene biosynthesis and perception, respectively, are presently used in apples to regulate preharvest fruit drop [3,18,19,20]. Ethylene biosynthesis results from the conversion of S-adenosyl-L-methionine (SAM) to 1-aminocyclopropane-1-carboxylate (ACC) via the action of ACC synthase (ACS) followed by the oxidation of ACC to ethylene, via ACC oxidase (ACO) [21,22]. Once ethylene is synthesized, it is perceived by a family of copper-binding membrane-associated receptors with ethylene-binding capacity [23]. There are several ethylene receptors that have been reported in apple fruit, including *MdERS1*, *MdERS2*, *MdETR1*, *MdETR2*, *MdETR5* [18,24,25,26,27]. Constitutive triple response 1 (*MdCTR1*) is a Raf-like serine/threonine-protein kinase acting as a key regulator of ethylene responses downstream of the ethylene receptors [28,29]. The plant growth regulator AVG impedes the activity of ACS, the rate-limiting step in ethylene biosynthesis [3,20,21,30,31], while 1-MCP binds to ethylene receptors, inactivating them and preventing the perception of ethylene and downstream signaling [9,19,32,33]. Although 1-MCP mainly prevents ethylene from binding to its receptors, it has been shown that it can reduce ethylene biosynthesis, thus boosting its action [18]. It has been reported that AVG and 1-MCP application timings, concentrations, as well as cultivars and locations in which they are used can impact their efficacy in controlling preharvest fruit drop. Previous work has documented that AVG and 1-MCP applications have displayed significant reductions in preharvest fruit drop in ‘Gala’ [9,34], ‘Honeycrisp’ [2,35], ‘Delicious’ [18,36], and ‘McIntosh’ [37] grown in different locations. However, studies evaluating and comparing AVG and 1-MCP effects on preharvest fruit drop, ethylene production rates, as well as ethylene-associated gene expression are lacking for ‘Honeycrisp’ fruit grown in the US mid-Atlantic.

Fruit quality results from several permanent changes in texture, flavor, and color that occur as the fruit ripens on-the-tree [38,39,40]. Utilization of AVG and 1-MCP plant growth regulators may significantly affect apple fruit ripening and thus fruit quality characteristics. It has been shown that AVG or 1-MCP applications can delay apple fruit flesh softening and starch breakdown [1,9,18,41,42,43,44,45]. However, for sugar contents and acidity, there are discrepancies in the effects of AVG and 1-MCP applications, as some studies report no effects [41,44,46], while others show that these plant growth regulators maintain higher titratable acidy and lower soluble solids content [2,9,35,36,45,47,48,49]. Furthermore, regarding fruit coloration, the responses seem to be different when treating fruit with AVG or 1-MCP, as the former has been reported to delay red skin color significantly with respect to the latter in cultivars such as ‘Gala’ [9,45] and ‘Cripps Pink’ [44], while in ‘Bisbee Delicious’, authors have reported no effects of these plant growth regulators on coloration [18]. This is of central importance in ‘Honeycrisp’ apples, as 50% blush skin coverage is required to qualify for premium-grade fruit packing [35,50,51]. Red skin coloration in apple fruit is majorly determined by the concentration of the pigment anthocyanin [52]. Anthocyanins are a product of the phenylpropanoid pathway [53], which begins with the precursor phenylalanine, and includes multiple enzymes throughout the pathway, such as phenylalanine ammonia-lyase (PAL), chalcone synthase (CHS), chalcone isomerase (CHI), flavanone 3-hydroxylase (F3H), dihydroflavonol 4-reductase (DFR), leucoanthocyanidin dioxygenase (LDOX), and UDP glucose-flavonoid 3-O-glucosyltransferase (UFGT) [54]. Ethylene production, among other factors, has been shown to significantly impact the biosynthesis of anthocyanins [30,31,55,56,57,58,59,60,61], therefore it is crucial to understand the effect of ethylene-related plant growth regulators on these pathways. 

Investigations of the effects of AVG and 1-MCP on apple preharvest fruit drop and fruit quality characteristics under the US mid-Atlantic environmental conditions are currently limited, despite previous research reporting the responses to these horticultural practices in other locations and climates. Furthermore, few studies have examined the effect of these ethylene-related plant growth regulators on ethylene production and on ethylene- and anthocyanin-related gene expression. To our knowledge, this has yet to be conducted with the cultivar ‘Honeycrisp’ in the US mid-Atlantic. Based on the above, this work has two main objectives: one, to characterize and compare the effects of AVG and 1-MCP applications on preharvest fruit drop, ethylene production rates, fruit quality properties, skin color, as well as on the transcript accumulation of ethylene biosynthesis and perception-related genes and of anthocyanin-biosynthesis-related genes in ‘Honeycrisp’ apples throughout ripening on-the-tree; and two, to identify significant correlations amongst all the evaluated parameters via multivariate analysis. 

## 2. Results

### 2.1. AVG and 1-MCP Effects on ‘Honeycrisp’ Fruit Drop during On-the-Tree Ripening 

**Figure 1:**
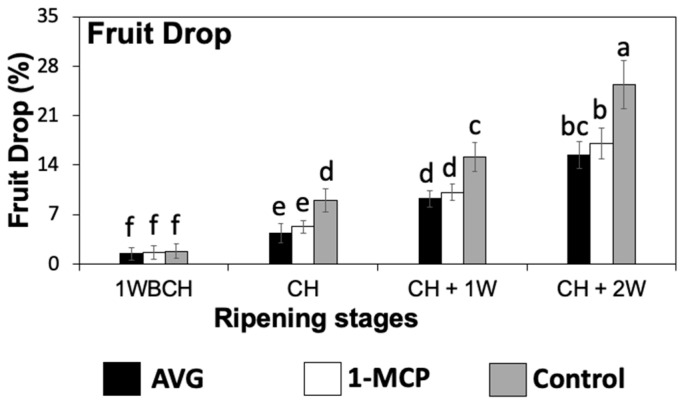
AVG and 1-MCP effects on ‘Honeycrisp’ preharvest fruit drop in Aspers, PA. Fruit drop was measured 1 week before commercial harvest (1WBCH), at commercial harvest (CH), 1 week after CH (CH + 1W), and 2 weeks after CH (CH + 2W). Values are means ± standard error. Different letters indicate significant differences (*p* ≤ 0.05) according to Tukey’s HSD test.

At 1 week before commercial harvest (1WBCH), there were no significant differences amongst treatments in fruit drop percentage, but preharvest fruit drop values began to rise remarkably from this period forward for all the assessed treatments (Figure 1). Treatments that received AVG and 1-MCP applications exhibited similar levels of fruit drop, reducing fruit drop ~1.5 times with respect to the control fruit from commercial harvest (CH) onwards. At 2 weeks after CH (CH + 2W), the control fruit presented fruit drop values of ~25% while AVG- and 1-MCP-treated fruit displayed values ranging between 15 and 17%. 

### 2.2. AVG and 1-MCP Effects on Ethylene Production and Physicochemical Parameters of ‘Honeycrisp’ during On-the-Tree Ripening and AVG and 1-MCP Effects on ‘Honeycrisp’ Fruit Drop during On-the-Tree Ripening 

The ethylene production rate significantly increased for all treatments throughout the assessed ripening stages, with CH + 2W displaying the highest values for this parameter for each treatment (Table 1). When comparing within each ripening stage, at CH and CH + 1W, the highest ethylene production rates were observed for the control fruit, followed by 1-MCP-treated fruit, and finally by AVG-treated fruit with the significantly lowest values. At CH + 2W, the control and 1-MCP-treated fruit exhibited no differences between them, while the AVG-treated fruit displayed the lowest ethylene production rates.

Regarding fruit weight, no significant differences were observed throughout all the evaluated ripening stages on-the-tree nor amongst treatments in this study (data not shown).

A significant reduction was detected in fruit flesh firmness during ripening on-the-tree for all treatments, although the control fruit displayed no differences between CH + 1W and CH + 2W (Table 1). For CH and CH + 1W, fruit flesh firmness values were significantly the highest for AVG-treated fruit, followed by 1-MCP-treated fruit, while the lowest values were obtained for the control fruit. At CH + 2W, AVG-treated fruit continued to exhibit the highest flesh firmness (>60 N), while 1-MCP-treated fruit and the control fruit showed no differences between them (<60 N). 

Starch pattern index was also affected by the different plant growth regulator treatments and evaluation periods (Table 1). As the ripening stages advanced, SPI significantly increased (implying a starch content decrease) for all treatments. At CH and CH + 1W, SPI values were significantly the highest for the control fruit, followed by 1-MCP-treated fruit and subsequently by AVG-treated fruit, which displayed the lowest values. For CH + 2W, the control and 1-MCP-treated fruit did not differ between them and presented significantly higher SPI values (SPI > 7.5) than the AVG-treated fruit (SPI = 6.5). 

In the case of SSC, our results show a significant rise in SSC values as the fruit ripened on-the-tree, for all the evaluated treatments (Table 1), although SSC values were statistically constant between CH + 1W and CH + 2W in all cases. Within treatments, significant differences were only observed at CH between AVG-treated fruit and the control fruit, with SSC values of <13% and >13%, respectively. 

Regarding the values of titratable acidity, these showed a significant reduction throughout the different assessed ripening stages for all treatments (Table 1). Within ripening stages, at CH and CH + 1W, TA values were significantly the highest for AVG-treated fruit, followed by 1-MCP-treated fruit, while the significantly lowest values were obtained for the control fruit. In the case of CH + 2W, AVG-treated fruit continued to exhibit the highest TA values (~0.40), while 1-MCP-treated fruit and the control fruit showed no differences between them (~0.30). 

### 2.3. AVG and 1-MCP Effects on ‘Honeycrisp’ Skin Color during On-the-Tree Ripening

Surface skin hue angle values were reduced (implying an increased red skin color) for fruit from all treatments as on-the-tree ripening advanced (Table 2). Values for surface skin hue angle were statistically highest for AVG-treated fruit, and significantly differed from 1-MCP-treated fruit and the control fruit that displayed no differences between them, at all ripening stages (Figure S1). Skin blush percentage also showed a significant increase as ripening advanced for all treatments. AVG-treated fruit was the exception, presenting no significant change in blush percentage from CH to CH + 1W (Table 2). Moreover, for all evaluation periods, the consistently lowest skin blush percentage was observed for AVG-treated fruit (35–49%), while 1-MCP-treated fruit and the control did not differ between them and presented skin blush percentage values between 43 and 60%. Remarkably, 1-MCP-treated fruit and the control fruit reached > 50% blush at CH + 1W, but AVG-treated fruit only reached ~50% blush in the last ripening stage (i.e., when evaluated at CH + 2W) (Table 2). 

Regarding background skin hue angle and index of absorbance difference (I_AD_), a significant reduction was observed in their values (implying a skin color change from green to yellow and a skin chlorophyll content reduction, respectively) as fruit ripened on-the-tree for all treatments (Table 2). In general, the values for background skin hue angle and I_AD_ were significantly the highest for AVG-treated fruit, and statistically differed from 1-MCP-treated fruit and the control fruit that displayed no differences between them for all assessed ripening stages.

### 2.4. AVG and 1-MCP Effects on the Expression of Ethylene-Related Genes in ‘Honeycrisp’ Fruit Flesh during On-the-Tree Ripening

#### 2.4.1. Ethylene-Biosynthesis-Related Genes

**Figure 2:**
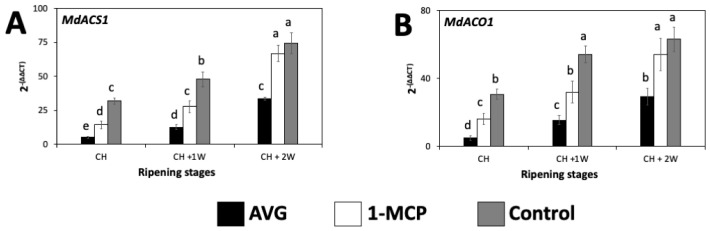
AVG and 1-MCP impacts on the relative expression of ethylene-biosynthesis-related genes of ‘Honeycrisp’ apples throughout ripening on-the-tree in Aspers, PA. (**A**) *MdACS1*, (**B**) *MdACO1*. Apples were assessed at commercial harvest (CH), 1 week after CH (CH + 1W), and 2 weeks after commercial harvest (CH + 2W). Values are means ± standard error. Different letters indicate significant differences (*p* ≤ 0.05) according to Tukey’s HSD test. 1-aminocyclopropane-carboxylase synthase (ACS), 1-aminocyclopropane-carboxylase oxidase (ACO).

The expression of ethylene-biosynthesis-related genes, *MdACS1* and *MdACO1*, exhibited a significant surge from CH to CH + 2W for all treatments (Figure 2), although for the control fruit, *MdACO1* expression was not significantly different between CH + 1W and CH + 2W (Figure 2B). When comparing within each ripening stage, at CH and CH + 1W, the highest transcript accumulation for *MdACS1* and *MdACO1* was observed for the control fruit, followed by 1-MCP-treated fruit, and finally by AVG-treated fruit with the significantly lowest values. At CH + 2W, the control and 1-MCP-treated fruit exhibited no differences between them, while AVG-treated fruit presented with the lowest gene expression levels (Figure 2).

#### 2.4.2. Ethylene-Perception-Related Genes

**Figure 3:**
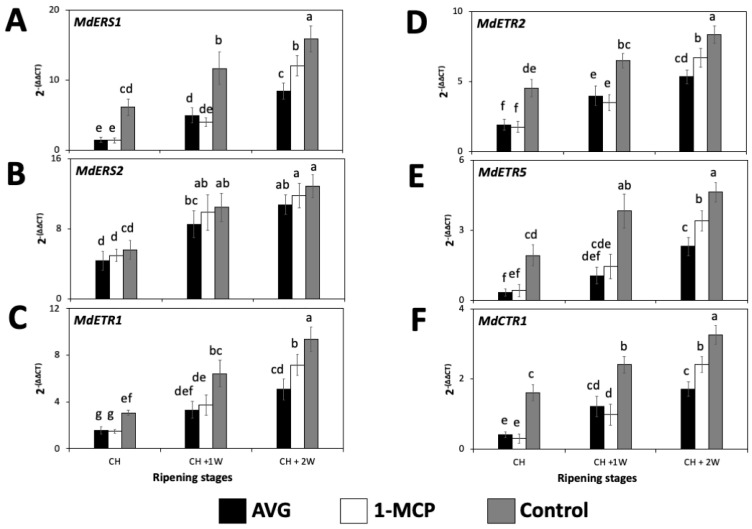
AVG and 1-MCP impacts on the relative expression of ethylene-perception-related genes of ‘Honeycrisp’ apples throughout ripening on-the-tree in Aspers, PA. (**A**) *MdERS1*, (**B**) *MdERS2*, (**C**) *MdETR1*, (**D**) *MdETR2*, (**E**) *MdETR5*, (**F**) *MdCTR1*. Apples were assessed at commercial harvest (CH), 1 week after CH (CH + 1W), and 2 weeks after commercial harvest (CH + 2W). Values are means ± standard error. Different letters indicate significant differences (*p* ≤ 0.05) according to Tukey’s HSD test. Ethylene-response sensor (ERS), ethylene receptor-type (ETR), constitutive triple response (CTR).

Transcript accumulation of all the assessed ethylene-perception-related genes showed a significant increase throughout the three assayed ripening stages for all the evaluated treatments (Figure 3). When comparing within each ripening stage, at CH and CH + 1W, the statistically highest transcript accumulation for *MdERS1*, *MdETR1*, *MdETR2*, *MdETR5*, and *MdCTR1* was observed for the control fruit, followed by 1-MCP and by AVG-treated fruit which displayed no significant differences between them (Figure 3). At CH + 2W, the highest transcript accumulation for *MdERS1*, *MdETR1*, *MdETR2*, *MdETR5*, and *MdCTR1* was observed for the control fruit, followed by 1-MCP-treated fruit and finally by AVG-treated fruit with the significantly lowest values. In the case of *MdERS2*, there were no significant differences amongst treatments within any of the three assessed ripening stages (Figure 3).

### 2.5. AVG and 1-MCP Effects on the Expression of Anthocyanin-Biosynthesis-Related Genes in ‘Honeycrisp’ Fruit Skin during On-the-Tree Ripening 

**Figure 4:**
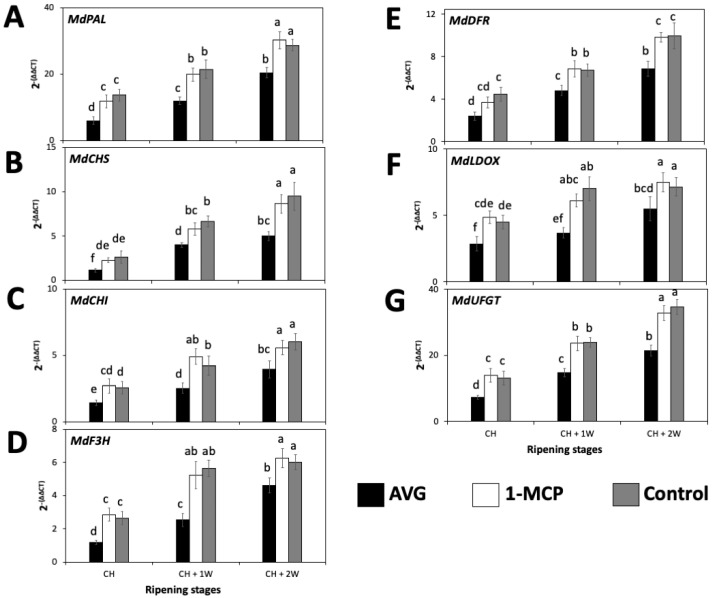
AVG and 1-MCP impacts on the relative expression of anthocyanin-biosynthesis-related genes of ‘Honeycrisp’ apples throughout ripening on-the-tree in Aspers, PA. (**A**) *MdPAL*, (**B**) *MdCHS*, (**C**) *MdCHI*, (**D**) *MdF3H*, (**E**) *MdDFR*, (**F**) *MdLOX*, (**G**) *MdUFGT*. Apples were assessed at commercial harvest (CH), 1 week after CH (CH + 1W), and 2 weeks after commercial harvest (CH + 2W). Values are means ± standard error. Different letters indicate significant differences (*p* ≤ 0.05) according to Tukey’s HSD test. Phenylalanine ammonia-lyase (PAL), chalcone synthase (CHS), chalcone isomerase (CHI), flavanone 3-hydroxylase (F3H), dihydroflavonol 4-reductase (DFR), leucoanthocyanidin dioxygenase (LDOX), UDP glucose-flavonoid 3-O-glucosyltransferase (UFGT).

Transcript accumulation of all assessed anthocyanin-biosynthesis-related genes consistently and significantly increased from CH to CH + 2W for all treatments (Figure 4). For 1-MCP-treated fruit and the control fruit, no differences were observed in gene expression of *MdCHI*, *MdF3H*, and *MdLDOX* between CH + 1W and CH + 2W ripening stages (Figure 4C,D,F). Additionally, within each ripening stage, the seven assessed key anthocyanin-biosynthesis-related genes consistently showed the significantly lowest transcript accumulation for AVG-treated fruit, while 1-MCP-treated fruit and the control fruit did not present differences between them and displayed expression profiles that were statistically higher than AVG-treated fruit in all cases (Figure 4). 

### 2.6. Associations between Fruit Drop, Ethylene Production, Physicochemical Properties, Skin Color, and Expression of Key Anthocyanin- and Ethylene-Related Genes in ‘Honeycrisp’ Fruit

**Figure 5:**
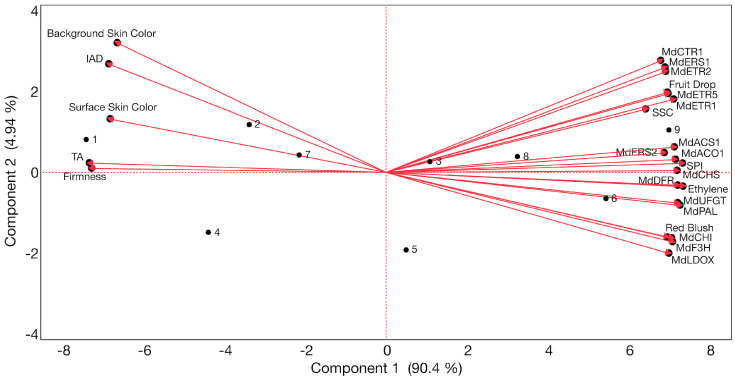
Principal component analysis of data acquired from fruit drop, ethylene production, physicochemical properties, skin color, expression of ethylene-biosynthesis-related and perception-related genes, as well as anthocyanin-biosynthesis-related genes of ‘Honeycrisp’ apples subjected to AVG and 1-MCP treatments and evaluated throughout ripening on-the-tree. Numbers correspond to the different treatments and evaluation periods/ripening stages that were examined (1 (AVG_CH), 2 (AVG_CH + 1W), 3 (AVG_CH + 2W), 4 (1-MCP_CH), 5 (1-MCP_CH + 1W), 6 (1-MCP_CH + 2W), 7 (Control_CH), 8 (Control_CH + 1W), 9 (Control_CH + 2W)). Starch pattern index (SPI), soluble solids content (SSC), titratable acidity (TA), index of absorbance difference (I_AD_). Codes for genes are defined in Figure 2, Figure 3 and Figure 4.

An estimation of Pearson correlation coefficients (Table S1) and a principal component analysis (PCA; Figure 5) were conducted including all the evaluated features described above for ‘Honeycrisp’ fruit in this work. 

Fruit drop displayed positive correlations with ethylene production (r = 0.92), SSC (r = 0.87), SPI (r = 0.94), skin blush (r = 0.83), with all the anthocyanin-biosynthesis-related genes (r ≥ 0.80), as well as with all the ethylene-biosynthesis-related (r ≥ 0.90) and perception-related genes (r ≥ 0.91). Contrariwise, fruit drop presented with negative correlations with TA (r = −0.92), flesh firmness (r = −0.90), I_AD_ (r = −0.78), as well as with surface and background skin hue angles (r = −0.77 and r = −0.73, respectively) (Table S1).

Ethylene production exhibited positive associations with SSC (r = 0.87), SPI (r = 0.99), skin blush (r = 0.94), with all the anthocyanin-biosynthesis-related genes (r ≥ 0.95), as well as with all the ethylene-biosynthesis-related (r ≥ 0.94) and perception-related genes (r ≥ 0.89); however, it revealed negative associations with TA (r = −0.99), flesh firmness (r = −0.99), IAD (r = −0.94), and surface and background skin hue angles (r = −0.92 and r = −0.91, respectively).

Flesh firmness showed positive correlations with TA (r = 0.98), IAD (r = 0.93), as well as with surface and background skin hue angles (r = 0.92 and r = 0.89, respectively) (Table S1). On the other hand, it exhibited negative correlations with SSC (r = −0.85), SPI (r = −0.99), skin blush (r = −0.95), with all the anthocyanin-biosynthesis-related genes (r ≤ −0.91), as well as with all the ethylene-biosynthesis-related (r ≤ −0.95) and perception-related genes (r ≤ −0.89).

The parameter of SPI positively correlated with SSC (r = 0.86), skin blush (r = 0.94), with all the anthocyanin-biosynthesis-related genes (r ≥ 0.92), as well as with all the ethylene-biosynthesis-related (r = 0.96) and perception-related genes (r ≥ 0.91). Moreover, SPI negatively correlated with TA (r = −0.99), I_AD_ (r = −0.91), and with surface and background skin hue angles (r = −0.89 and r = −0.88, respectively) (Table S1). For SSC, there was a positive association with skin blush (r = 0.73), with all the anthocyanin-biosynthesis-related genes (r ≥ 0.74), as well as with all the ethylene-biosynthesis-related (r = 0.75) and perception-related genes (r ≥ 0.82); however, SSC negatively associated with TA (−0.83), I_AD_ (r = −0.72), and with surface and background skin hue angles (r = −0.83 and r = −0.65, respectively). Conversely, TA presented positive correlations with I_AD_ (r = 0.94) and skin hue angles (surface and background, r = 0.89 and r = 0.90, respectively), while displaying significant negative correlations with fruit skin blush (r = −0.94), with all the anthocyanin-biosynthesis-related genes (r ≤ −0.94), as well as with all the ethylene-biosynthesis-related (r ≤ −0.95) and perception-related genes (r ≤ −0.90) (Table S1).

Concerning surface and background skin hue angles as well as IAD, these were all positively associated with one another (r ≥ 0.90); however, they displayed negative associations with skin blush (r ≤ −0.91), all the anthocyanin-biosynthesis-related genes (r ≤ −0.86), as well as with all the ethylene-biosynthesis-related (r ≤ −0.84) and perception-related genes (r ≤ −0.67). Conversely, skin blush exhibited positive correlations with all the anthocyanin-biosynthesis-related genes (r ≥ 0.87), as well as all the ethylene-biosynthesis-related (r ≥ 0.92) and perception-related genes (r ≥ 0.79).

The anthocyanin-biosynthesis-related genes displayed positive associations with each other (r ≥ 0.91), as well as with all the ethylene-biosynthesis-related (r ≥ 0.87) and perception-related genes (r ≥ 0.78). Furthermore, all the assessed ethylene-biosynthesis-related genes positively associated with each other (r ≥ 0.97), as well as with all the ethylene-perception-related genes (r ≥ 0.83). The latter also presented positive correlations amongst them (r ≥ 0.83) (Table S1).

Regarding the PCA, the results showed that the total variation (95.34%) was explained by the first (90.4%) and by the second (4.94%) principal components (Figure 5). The distribution of the three treatments and the assessed ripening stages alongside the first principal component was defined by the background and surface skin hue angle, I_AD_, TA, and flesh firmness on the negative side of the axis (associated with AVG-treated fruit at CH and CH + 1W, with 1-MCP-treated fruit at CH, and with control fruit at CH) and by fruit drop, ethylene production, SPI, SSC, skin blush, as well as by all the evaluated anthocyanin-biosynthesis-related genes and all the assessed ethylene-biosynthesis-related and perception-related genes on the positive side of the axis (associated with AVG-treated fruit at CH + 2W, with 1-MCP-treated fruit at CH + 1W, and CH + 2W and with control fruit at CH + 1W and CH + 2W).

## 3. Discussion

Ethylene has been shown to strongly influence apple preharvest fruit drop [8,9,62]. This notion was supported by our results through the positive correlation displayed between fruit drop and ethylene production and agrees with work on ‘Golden Delicious’ apples demonstrating a linear increase in preharvest fruit drop with the rise in ethylene production [16]. In agreement with previous reports [18,36,63,64], the reduction in transcript accumulation of ethylene-biosynthesis-related and perception-related ethylene genes in ‘Honeycrisp’ apples treated with AVG and 1-MCP throughout on-the-tree ripening can explain the lower fruit drop rates observed with these treatments. Furthermore, AVG and 1-MCP applications, with similar timings and concentrations as used in our work, have been shown to reduce preharvest fruit drop in several apple cultivars, such as ‘Gala’ [9,34], ‘Honeycrisp’ [2,35], ‘Delicious’ [18,36], and ‘McIntosh’ [37] in different regions, supporting our results.

Apples display a climacteric fruit behavior, and thus increase ethylene production and respiration rates during ripening [12,13]. This is known to be driven by the autocatalytic System-2 ethylene biosynthesis model [65,66]. Furthermore, *MdACS1* has been shown to be the rate-limiting step for System-2 ethylene biosynthesis during apple ripening [27,67]. Hence, the significant decrease in *MdACS1* and consequently in *MdACO1* transcript accumulation in AVG- and 1-MCP-treated ‘Honeycrisp’ fruit, as compared to control, at CH and CH + 1W, can explain the paralleled lower ethylene production rates obtained in this work for both treatments, in agreement with previous studies [25,30,64,68]. Moreover, the significantly lower expression levels of ethylene-biosynthesis-related genes observed in fruit treated with AVG as compared to 1-MCP throughout ripening can be supported by their different modes of action, as AVG impedes the activity of ACS, while 1-MCP is an inhibitor of ethylene binding to its receptors [9,19,20] which consequently prevents downstream signaling and thus results in the reduction of the expression of genes associated with ethylene biosynthesis by impacting the autocatalytic ethylene production [69,70]. 

Regarding ethylene perception, ethylene receptors have been reported to act as negative regulators of ethylene; therefore, an increased presence of receptors results in decreased sensitivity to ethylene [22,71]. The binding of ethylene to receptors elicits receptor protein breakdown, hence reducing overall receptor protein levels and thus allowing the ethylene response [72]. In this study, the transcript levels of genes encoding for ethylene receptors were significantly decreased by AVG and 1-MCP treatments with respect to the control (*MdERS1*, *MdETR1*, *MdETR2*, *MdETR5*). It could be assumed that the binding of 1-MCP to ethylene receptors might result in decreased receptor protein breakdown, consequently blocking the ethylene response. The lack of direct correspondence between ethylene-receptor-related gene expression and ethylene production rates at CH + 2W for 1-MCP-treated and control fruit could be explained by receptor-related genes not being the rate-limiting step in ethylene production or moreover by the occurrence of posttranscriptional transcript regulation and/or even posttranslational modifications at the enzymatic level. *MdERS2*, remarkably, did not exhibit any differences in transcript accumulation amongst treatments, which is consistent with previous studies in ‘Delicious’ apples that showed that AVG and 1-MCP did not affect its expression [64,68]. Furthermore, *MdCTR1*, which has also been reported to act as an ethylene negative regulator, is known to interact with ethylene receptors downstream [73]. In the present work, AVG and 1-MCP treatments significantly decreased the transcript accumulation of *MdCTR1* as compared to the control, supporting the notion that *MdCTR1* seems to be induced by ethylene. These findings correspond with what was observed in tomato for *LeCTR1* [74], as well as in previous works in apple [33] and plum [75]. Likewise, the increased expression levels of *MdCTR1* during ‘Honeycrisp’ fruit ripening on-the-tree for all treatments in this study is in agreement with what has been reported before for members of the Rosaceae family such as apple [25,33], plum [75,76], and pear [77]. It has been suggested that the promotion of ethylene induced by a negative regulator of ethylene response during ripening could be the means to moderate the significant rise in ethylene production and hence control the ripening process [78]. 

Ethylene is known to play a primary role in fruit ripening [12,13,39,40]; therefore, practices that control preharvest fruit drop by altering ethylene biosynthesis (AVG) or perception (1-MCP) are anticipated to have an impact on fruit maturity. In fact, at CH and CH + 1W, both AVG and 1-MCP delayed flesh fruit softening as well as starch breakdown as compared to the control, hence decreasing fruit overripening if the fruit is left hanging on the tree, consistent with previous reports in different apple cultivars [9,18,41,42,43,44,45]. In the present work, AVG and 1-MCP treatments maintained higher titratable acidity than the control fruit, in agreement with other studies [2,35,45,48,49], while soluble solids contents were lower with AVG at CH, consistent with previous works [9,36,47]. However, other reports have shown that preharvest AVG and 1-MCP applications have no effect on acidity nor soluble solids contents [41,44,46]. These differences in studies can potentially be attributed to variability in climates, preharvest management practices, evaluated apple cultivars, as well as to the timing and concentration of the applications. Regarding fruit weight, the lack of differences amongst treatments has been previously reported [1,9,36,79], supporting the notion that weight is not affected by ethylene. In general, AVG was more effective than 1-MCP in delaying fruit maturity at all evaluation periods. The latter could be a result of the significantly lower ethylene production rates of AVG-treated fruit throughout ripening on-the-tree, which is in accordance with previous findings [42,45]. This is of critical importance as it implies that preharvest 1-MCP and AVG treatments could be extending the postharvest storage and shelf-life capacity of ‘Honeycrisp’ fruit, which is being presently investigated. 

As ethylene is also involved in the regulation of anthocyanin accumulation during ripening [58,59,60,61,80,81,82] and anthocyanins majorly determine the development of apple red skin coloration, the use of ethylene plant growth regulators is anticipated to impact this key fruit marketability-related property. This notion is supported by the positive associations reported in this work between ethylene production and skin blush as well as with anthocyanin-biosynthesis-related gene expression, and the negative correlations between ethylene production and skin hue angle. AVG-treated fruit has been shown to hinder red skin color significantly with respect to 1-MCP-treated fruit and/or control fruit in cultivars such as ‘Gala’ [9,45] and ‘Cripps Pink’ [44]. This is consistent with our results, where the lowest transcript accumulation for all assayed anthocyanin-biosynthesis-related genes was observed for AVG-treated fruit throughout ripening on-the-tree, and hence explains the delay of AVG-treated ‘Honeycrisp’ fruit to reach the minimum required 50% skin blush coverage, which was only attained at CH + 2W. The different modes of action of AVG and 1-MCP could explain the significant differences in ‘Honeycrisp’ red skin color obtained in this study between the treatments. It has been reported that there is a positive interaction between *MdMYB10*, a transcription factor which regulates the expression of anthocyanin-biosynthesis-related genes, and *MdACS1* and *MdACO1* [59,83,84,85]. As AVG directly impedes the activity of ACS, but 1-MCP instead represses ethylene perception [19,20], this could explain why the former presents the most significant delay in red skin color development. 

The differences in the placement of the different treatments/ripening stages assessed in this work in the first principal component of the PCA can be explained by the AVG-treated fruit displaying the lowest fruit drop, ethylene production, ethylene biosynthesis, and perception-related gene expression, most delayed fruit maturity, most inhibited red skin color (only reaching the minimum required 50% blush at CH + 2W), as well as most downregulated anthocyanin-biosynthesis-related gene expression, at all evaluation periods. Furthermore, 1-MCP-treated fruit followed, also showing the lowest fruit drop, but at the same time, exhibiting an intermediate positioning concerning ethylene production, transcript accumulation of ethylene-biosynthesis-related and perception-related genes, fruit maturity, as well as a fruit blush that reached the minimum required 50% at CH + 1W (supported by 1-MCP-treated fruit exhibiting the highest expression of anthocyanin-biosynthesis-related genes at all stages). Finally, the control fruit exhibited the significantly highest fruit drop, ethylene production, ethylene-related gene expression, and most advanced fruit maturity (i.e., increasing overripening), while promoting skin blush (reaching the minimum required 50% at CH + 1W) and transcript accumulation of anthocyanin-biosynthesis-related genes at all evaluation periods. Future work is ongoing to assess the effects of these preharvest plant growth regulators on other economically important apple cultivars.

## 4. Materials and Methods

### 4.1. Plant Material and Treatments 

This work was conducted in a block of 12-year-old ‘Honeycrisp’ trees (*Malus domestica* Borkh.) grafted on M9 rootstock and situated in Aspers, PA. Trees were trained to a central leader and spaced at 1.5 × 4 m. Three treatments were established including the plant growth regulator AVG (ReTain, Valent Biosciences Corporation, Libertyville, IL, USA) [applied four weeks before the anticipated commercial harvest date at 130 mg a.i. L^−1^], the plant growth regulator 1-MCP (Harvista 1.3 SC, Agrofresh, Philadelphia, PA, USA) [applied when the starch pattern index (SPI) was 3 at 150 mg a.i. L ^−1^], as well as an untreated control, in the 2022 season. A randomized complete block design was used to establish the three treatments, each with four replications of thirty trees. All sprays were performed using a pressurized orchard sprayer and were mixed with 1.0 mL L^−1^ Silwet-77 organosilicone surfactant. For 1-MCP application, an Agrofresh formulation tank, injection pump, and calibration tube were mounted to the sprayer. 

During the season, maturity indices were examined [35] in order to harvest fruit at the optimum commercial maturity, using the control fruit as a reference. Evaluation periods included three on-the-tree ripening stages: commercial harvest (CH), 1 week after CH (CH + 1W), and 2 weeks after CH (CH + 2W). For each evaluation date, each of the four replications per treatment comprised thirty fruit per replication. After each harvest, the fruit was immediately transported to the laboratory. Five fruit samples per replication were used for the assessment of ethylene production rate, and these were also washed, peeled (skin tissue), and cut into small pieces (flesh tissue). Each tissue type was pooled together, frozen, and homogenized in liquid nitrogen, and stored at −80 °C for further analyses. The remaining twenty-five fruit samples per replication were used to examine quality-related physicochemical properties.

### 4.2. Fruit Drop Assessments

Fruit drop measurements were conducted as previously described [30]. In summary, for each replication within each treatment, a total of five limbs with twenty fruit each, were labeled from alternate sides of the trees and from different trees, two weeks before the predicted CH. Starting from one week before CH (1WBCH) until 2 weeks after CH (CH + 2W), the labeled fruit that remained on each limb was counted each week. The fruit drop percentage was assessed as compared to the initial number of fruit on each limb.

### 4.3. Ethylene Production

A static system was used to assess fruit ethylene production rates (μL C_2_H_4_ kg^−1^ h^−1^). Each fruit was incubated in a 1 L airtight jar fitted with rubber septa at 20 °C for 1 h. Ethylene production was determined by assessing ethylene concentration in the gas phase of the jars, determined by removing a 1 mL headspace gas sample from each jar and injecting it into a gas chromatograph (GC-2014C, Shimadzu Co., Kyoto, Japan) equipped with an activated alumina column attached to a flame ionization detector as previously described [35,86,87,88]. The temperatures for the injector, detector, and oven were correspondingly 140, 150, and 80 °C. 

### 4.4. Fruit Physicochemical and Skin Color Evaluations

Fruit weight, skin color, index of absorbance difference (I_AD_), blush percentage, fruit flesh firmness, starch pattern index (SPI), soluble solids content (SSC), and titratable acidity (TA) were assessed as previously described [35,39]. In summary, for skin color, evaluations of the surface and background color were accomplished via a colorimeter (Konica Minolta CR400 Chroma Meter, Konica Minolta Sensing, Inc., Osaka, Japan) on two opposed sides of each fruit. Hue angle (hue°) was calculated as described before [89]. A Delta Absorbance (DA) Meter (TR Turoni, Forli, Italy) was used to measure the index of absorbance difference (I_AD_) by averaging the measurements performed at three different points on the surface of each fruit [90]. A TA.XT Plus Connect texture analyzer (Texture Technologies Corp., Scarsdale, NY, USA) equipped with a 50 kg loadcell and analyzed with the Exponent TE32 (v6.0, Texture Technologies Corp., Scarsdale, NY, USA) software fitted with an 11.1 mm diameter probe, was used to measure fruit flesh firmness in the two opposed peeled sides (the peeled portion was about 2 mm thick) of each fruit over a distance of 8 mm at a speed of 8 mm s^−1^. The Cornell generic chart, where 1 and 8 represent 100% and 0% stained starch, respectively [91], was used to determine the values for SPI of each fruit cut at the equator. SSC values were measured using a digital hand-held refractometer (Atago, Tokyo, Japan), while TA was assessed by using an automatic titrator (855 Robotic Titrosampler; Metrohm, Riverview, FL, USA) [12,40]. 

### 4.5. Real-Time Quantitative RT-PCR Analysis

The cetyltrimethylammonium bromide (CTAB)/NaCl method [92] was used to isolate RNA from the apple skin and flesh following what was previously described [12,30,93]. First-strand complementary DNA synthesis, primer design, and quantitative PCR were performed based on previous reports [87]. Primer sets that were used in this study for the amplification of the target genes are indicated in Table 3. Relative gene expression analysis was conducted based on the comparative cycle threshold method [94] and the expression of actin (*MdACT*) was used as a reference gene.

### 4.6. Statistical Analysis

Generalized linear mixed models were used to model the response variables comprising treatments and evaluation periods (on-the-tree ripening stages) as fixed factors, and block as a random factor to define the statistical significance of the interactions and main effects (analysis of variance, ANOVA). If statistically significant differences were obtained, separation of means was carried out via Tukey’s HSD test at a 5% significance level.

Pearson’s correlation coefficients, using mean-centered data, were calculated for each pairwise combination of assessed features. To visualize the PCA, a ‘biplot’ graph was used, indicating the relationships among variables (fruit drop, ethylene production, physicochemical properties, skin color, gene expression values) and the assessed treatments and evaluation periods (on-the-tree ripening stages). To define the number of principal components that allowed capturing most of the variation, the Scree test was used. All the statistical analyses were performed using the software package JMP (ver 15.2, SAS Institute, Cary, NC, USA).

## 5. Conclusions

‘Honeycrisp’ apples subjected to different ethylene preharvest plant growth regulator treatments in the mid-Atlantic US and assessed throughout ripening on-the-tree revealed that both AVG and 1-MCP significantly and efficiently minimized preharvest fruit drop with respect to the control fruit. Furthermore, we demonstrated that AVG and 1-MCP applications had no effect on ‘Honeycrisp’ fruit weight, but the application of AVG was more effective in decreasing ethylene production, downregulating the expression of ethylene-biosynthesis-related and ethylene-perception-related genes, and delaying fruit maturity compared to 1-MCP. However, AVG negatively impacted apple red skin color and exhibited the lowest expression of anthocyanin-biosynthesis-related genes, only allowing apples to reach the minimum required 50% blush at the last evaluated ripening stage (CH + 2W). Furthermore, 1-MCP-treated fruit displayed an intermediate behavior between AVG-treated and control fruit, therefore decreasing ethylene production rates and the associated gene expression as well as delaying fruit maturity (i.e., decreasing overripening) when compared to the control fruit. Remarkably, 1-MCP did not sacrifice red skin color development or anthocyanin-biosynthesis-related gene expression, thus exhibiting >50% blush in parallel with the control fruit at CH + 1W. 

## Figures and Tables

**Table 1:** AVG and 1-MCP effects on ethylene production rates and physicochemical parameters of ‘Honeycrisp’ apples assessed throughout ripening on-the-tree in Aspers, PA.

Treatment	Ethylene (μL C_2_H_4_ kg^−1^ h^−1^)			Firmness (N)			SPI (1 to 8)			SSC (%)			TA (% Malic Acid)		
	CH	CH + 1W	CH + 2W	CH	CH + 1W	CH + 2W	CH	CH + 1W	CH + 2W	CH	CH + 1W	CH + 2W	CH	CH + 1W	CH + 2W
AVG	1.9 ± 0.5 f	11.5 ± 0.8 e	28.0 ± 1.9 c	79.9 ± 1.4 a	73.0 ± 1.1 b	63.8 ± 1.5 c	3.2 ± 0.1 f	4.8 ± 0.2 d	6.5 ± 0.4 b	12.7 ± 0.3 d	13.7 ± 0.2 ab	13.8 ± 0.3 a	0.53 ± 0.03 a	0.48 ± 0.01 b	0.39 ± 0.02 e
1-MCP	9.9 ± 1.8 e	24.3 ± 2.4 cd	36.6 ± 3.5 ab	74.4 ± 1.7 b	66.1 ± 1.1 c	58.0 ± 0.9 d	3.8 ± 0.2 e	5.8 ± 0.3 c	7.6 ± 0.4 a	12.9 ± 0.2 cd	13.6 ± 0.2 ab	13.8 ± 0.2 a	0.49 ± 0.01 b	0.42 ± 0.02 d	0.33 ± 0.02 f
Control	16.7 ± 2.0 d	32.1 ± 1.6 b	39.1 ± 3.2 a	67.1 ± 1.3 c	59.7 ± 1.7 d	57.2 ± 1.5 d	4.6 ± 0.2 d	6.8 ± 0.4 b	7.8 ± 0.5 a	13.3 ± 0.4 bc	13.9 ± 0.2 a	14.1 ± 0.3 a	0.45 ± 0.02 cd	0.38 ± 0.01 e	0.33 ± 0.01 f

Apples were harvested at commercial harvest (CH), 1 week after CH (CH + 1W), and 2 weeks after CH (CH + 2W). N, Newton; SPI, starch pattern index (1–8 scale); SSC, soluble solids content; TA, titratable acidity. Values are means ± standard error. Different letters indicate significant differences (*p* ≤ 0.05) according to Tukey’s HSD test.

**Table 2:** AVG and 1-MCP effects on ‘Honeycrisp’ fruit color evaluated throughout ripening on-the-tree in Aspers, PA.

Treatment	Surface Skin Color (Hue°)			Skin Blush (%)			Background Skin Color (Hue°)			Index of Absorbance Difference (I_AD_)		
	CH	CH + 1W	CH + 2W	CH	CH + 1W	CH + 2W	CH	CH + 1W	CH + 2W	CH	CH + 1W	CH + 2W
AVG	59.3 ± 2.6 a	48.9 ± 2.5 bc	47.3 ± 1.9 bc	35 ± 1.8 d	40 ± 2.9 d	49 ± 2.3 b	112.3 ± 3.7 a	110.2 ± 3.3 a	103.1 ± 2.8 bc	0.9 ± 0.04 a	0.8 ± 0.05 ab	0.6 ± 0.01 c
1-MCP	50.3 ± 2.6 b	41.7 ± 1.4 de	39.5 ± 1.0 de	43 ± 2.0 c	50 ± 1.9 b	58 ± 3.0 a	104.4 ± 2.7 bc	98.7 ± 2.9 de	96.4 ± 2.5 e	0.6 ± 0.03 c	0.5 ± 0.02 de	0.4 ± 0.01 ef
Control	46.8 ± 1.8 bc	38.5 ± 1.2 e	39.1 ± 0.8 e	44 ± 2.2 c	51 ± 2.4 b	60 ± 3.1 a	105.6 ± 3.1 b	100.9 ± 2.2 cd	95.1 ± 2.3 e	0.7 ± 0.03 bc	0.5 ± 0.01 de	0.4 ± 0.02 ef

Apples were harvested at commercial harvest (CH), 1 week after CH (CH + 1W), and 2 weeks after CH (CH + 2W). Values are means ± standard error. Different letters indicate significant differences (*p* ≤ 0.05) according to Tukey’s HSD test.

**Table 3:** Primers used in qRTPCR in ‘Honeycrisp’ fruit.

Gene Name	Description	Primer Orientation	Primer Sequence (5′ to 3′)
*MdPAL*	Phenylalanine ammonia-lyase	Forward Reverse	GTGCTGTGGAGTCCCCGCTT GGTGAGGCTCTCTCCGCCAAGT
*MdCHS*	Chalcone synthase	Forward	GGAGACAACTGGAGAAGGACTGGAA
		Reverse	CGACATTGATACTGGTGTCTTCA
*MdCHI*	Chalcone isomerase	Forward	GGGATAACCTCGCGGCCAAA
		Reverse	GCATCCATGCCGGAAGCTACAA
*MdF3H*	Flavanone 3-hydroxylase	Forward	TGGAAGCTTGTGAGGACTGGGGT
		Reverse	CTCCTCCGATGGCAAATCAAAGA
*MdDFR*	Dihydroflavonol 4-reductase	Forward	GATAGGGTTTGAGTTCAAGTA
		Reverse	TCTCCTCAGCAGCCTCAGTTTTCT
*MdLDOX*	Leucoanthocyanidin dioxygenase	Forward	CCAAGTGAAGCGGGTTGTGCT
		Reverse	CAAAGCAGGCGGACAGGAGTAGC
*MdUFGT*	UDP glucose-flavonoid 3- o -glucosyl transferase	Forward	CCACCGCCCTTCCAAACACTCT
		Reverse	CCACCGCCCTTCCAAACACTCT
*MdACS1*	1-aminocyclopropane-carboxylase (ACC) synthase	Forward	CTCCTCCTTTCCTTCGTTGA
		Reverse	ACCATGTCGTCGTTGGAGTAG
*MdACO1*	ACC oxidase	Forward	ATCAATGATGCTTGTGAGAACTG
		Reverse	GGTCTTCTTGTAGTGATCCTTGG
*MdERS1*	Ethylene-response sensor	Forward	TCCAGAACTGGTATGAACCTACA
		Reverse	AGAACTGTTGAAGACTTCGTTGA
*MdERS2*	Ethylene-response sensor	Forward	TGCGAAACCAGAATCTTCAAGA
		Reverse	CCTCAGTTGACGCTGGATAAAA
*MdETR1*	Ethylene receptor-type	Forward	GCACCTAGGATGTGATGTAACAG
		Reverse	TCATGTATACGGACAGCAAGTTC
*MdETR2*	Ethylene receptor-type	Forward	AGGCAAACAAAGGGATGACA
		Reverse	AGGCAAACAAAGGGATGACA
*MdETR5*	Ethylene receptor-type	Forward	GTTCTTCCGGTTGCAGATTC
		Reverse	ATGCATTGGCCTTCTCATTC
*MdCTR1*	Constitutive triple response	Forward	ACAAGATTTTCATGCCGAAC
		Reverse	TATGGACAAGTTTGGAGGCT
*MdACT*	Actin	Forward Reverse	TGACCGAATGAGCAAGGAAATTACT TACTCAGCTTTGGCAATCCACATC

## Data Availability

Data are contained in the article.

## Supplementary Materials

The following supporting information can be downloaded at: https://www.mdpi.com/article/10.3390/plants13172524/s1, Figure S1: AVG and 1-MCP effect on ‘Honeycrisp’ fruit skin coloration one week after commercial harvest in Aspers, PA. Table S1: Pearson correlation coefficients for preharvest fruit drop, ethylene production, physicochemical parameters, skin color, expression of ethylene-biosynthesis-related and perception-related genes, as well as anthocyanin-biosynthesis-related genes of ‘Honeycrisp’.
