# Supplementary figures and images for: Aminoethoxyvinylglicine and 1-Methylcyclopropene: Effects on Preharvest Drop, Fruit Maturity, Quality, and Associated Gene Expression of ‘Honeycrisp’ Apples in the US Mid-Atlantic
Source: Plants (Basel). 2024 Sep 8;13(17):2524. doi: 10.3390/plants13172524 (PMC11397464; doi:10.3390/plants13172524)

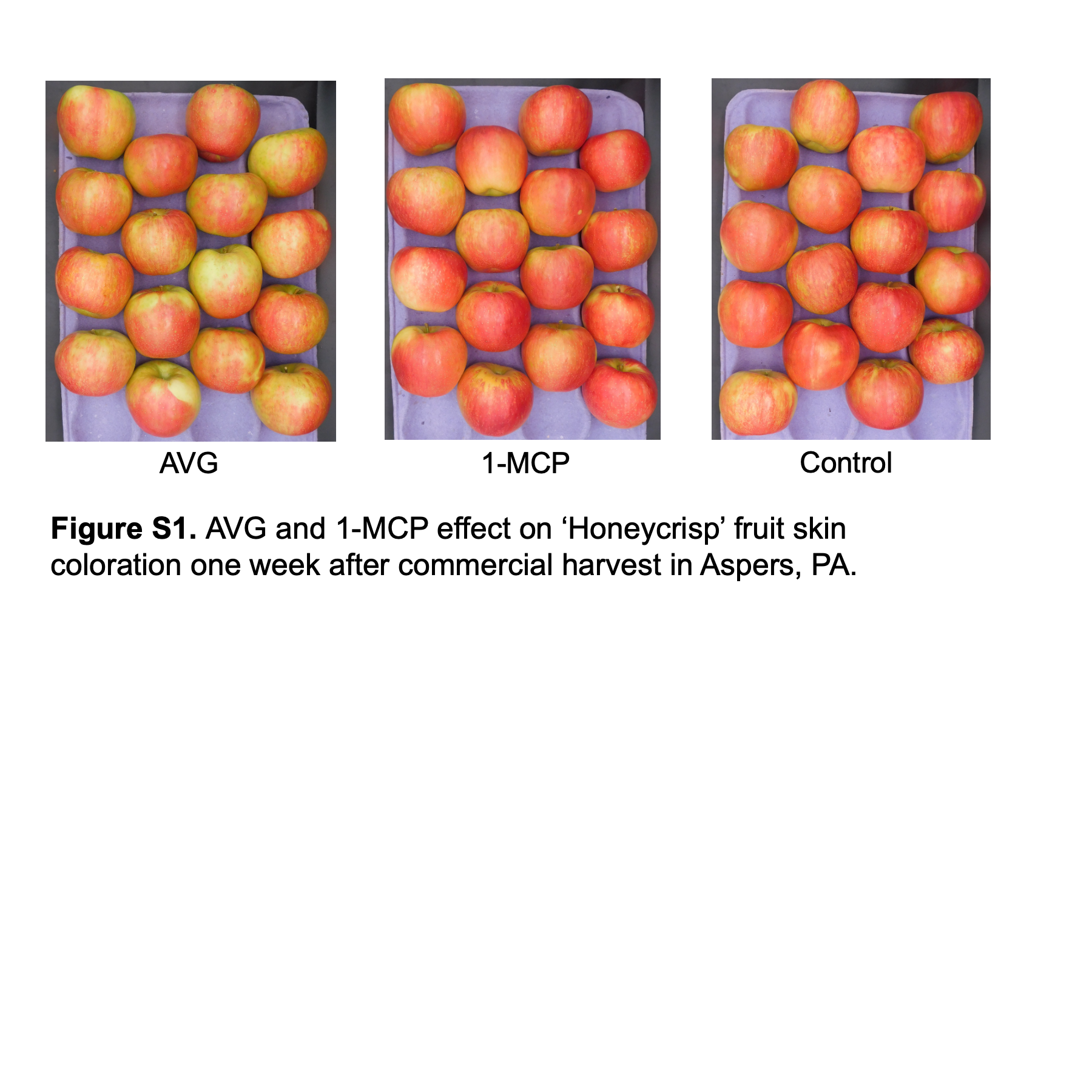

Supplement: Supplementary file 1 [file plants-13-02524-s001.zip › Figure S1.png]
